# Validity and reliability of the Amharic version of the Schwartz Center Compassionate Care Scale

**DOI:** 10.1371/journal.pone.0248848

**Published:** 2021-03-23

**Authors:** Merkeb Zeray, Damen Haile Mariam, Zekariyas Sahile, Alemayehu Hailu

**Affiliations:** 1 Department of General Public Health, School of Public Health, College of Health Sciences, Aksum University, Aksum, Ethiopia; 2 Department of Health Service Management, School of Public Health, College of Health Sciences, Addis Ababa University, Addis Ababa, Ethiopia; 3 Department of Public Health, College of Medicine and Health Science, Ambo University, Ambo, Ethiopia; 4 Department of Global Public Health and Primary Care Medicine, Bergen Center for Ethics and Priority Setting, The University of Bergen, Bergen, Norway; Iranian Institute for Health Sciences Research, ISLAMIC REPUBLIC OF IRAN

## Abstract

**Background:**

Compassionate care is the sensitivity shown by health care providers to understand another person’s suffering and a willingness to help and to promote the well being of that person. Although monitoring of compassionate care is key to ensuring patient-centered care, there is no validated tool in the Ethiopian context that can be applied to measure compassionate care. Therefore, this study aimed to assess the structural validity and reliability of the 12-item Schwartz Center Compassionate Care Scale^®^ (SCCCS) in the Ethiopian context.

**Methods:**

The structural validity and reliability of the 12-item Schwartz Center Compassionate Care Scale^®^ were investigated in a sample of 423 oncology patients in the adult Oncology department of Tikur Anbessa Specialized Hospital in Addis Ababa, Ethiopia. The internal consistency of the instrument was examined based on Cronbach’s alpha coefficient, and the structural validity was evaluated by subjecting the items of the instrument to factor analysis. Statistical analysis was made using SPSS version 23.0.

**Results:**

We have found that the Schwartz Center Compassionate Care scale is a two-factor structure (recognizing suffering and acting to relieve suffering). The scale has high overall scale reliability, which was 0.88, and subscale reliability of 0.84 for both recognizing suffering and acting to relieve suffering factors.

**Conclusions:**

The Schwartz Center Compassionate Care Scale has high internal consistency and acceptable structural validity value. The tool can be used to measure compassionate care practice in the Ethiopian context.

## Introduction

Compassion has a deep emotional sensitivity to human suffering, which requires a personal understanding of the suffering of others and caring for them in a way that brings comfort to the sufferer [1]. It is all about recognizing, understanding, and resonating emotionally with another’s concerns, distress, pain, or suffering coupled with an acknowledgment, motivation, and relational action to ameliorate them [2].

Compassionate and compassionate related care has resulted in alleviating pain, promoting fast recovery from acute illness, assisting in the management of chronic illness, relieving anxiety, and better resource management and reducing costs. Compassionate care also has physiological benefits such as altering heart rhythm [3–7].

To measure compassionate care accurately, it is necessary to have a robust and psychometrically validated instrument within a given setting. This is due to the Patient’s experience of compassionate medical care could vary across different cultures and religions, health professionals’ competency level, and the working health care environment [8].

Assessing compassionate care using tools developed in a different setting without validation; the tool might not show us the exact or the right compassionate health care status of a given service due to the aforementioned variations across settings. These reasons warrant not to assess the status of compassionate care without conducting a validation study in our context.

Different tools have been developed to measure compassionate care demonstration from the patient’s perspective. These include the 12-item Schwartz Center Compassionate Care Scale^®^ (SCCCS); used to measure patient perceptions of care provided by attending physician [8], the compassionate care assessment tool (CCAT); used to evaluate compassionate nursing care in acute hospital environments [9], patient compassion model (PCM); used to measure compassionate care among advanced cancer and non-cancer palliative patients [10]. None of the above compassionate care measuring tools have been assessed or examined for their validity or adapted and used to measure compassionate care in the Ethiopian context.

The SCCCS tool is considered a reliable and valid measure of patients’ perceptions of compassionate health care of physicians and the healthcare team’s. There is not any evidence that shows that the SCCCS works in languages other than English. The instrument was used to measure patients’ perceptions of compassionate health care of physicians and the healthcare team’s during a recent hospitalization. Patients completed the items of the tool using a ten-point scale from 1 (not at all successful) to 10 (very successful). The items were developed by a group of people from patients, family members, policymakers, and advocators and finally adapted through a focus group discussion with patients, physicians, and nurses [8, 11].

The generated items, which were initially 16 items, were assessed for validity and reliability in the USA among 800 recently hospitalized patients and 510 physicians through a national phone interview. The psychometric property analysis results showed that the SCCCS is a unidimensional compassionate care measuring tool with 12 items [8]. The SCCCS tool was applied in the Ireland setting and the reliability and validity analysis result was consistent with the USA’s findings [12].

SCCCS has been used in various settings for different purposes. To mention some, the tool was used to assess compassionate care practice in the USA [1], to assess the predictors of compassionate care in the USA [13], compare with other tools in Canada [14] and the UK [11], and to examine the reliability and validity of the tool in a study conducted in Ireland [12]. In two countries in the USA and Ireland, the validity and reliability of the tool were examined and the results showed that the tool is potentially useful in different settings for measuring compassionate care practice [8, 12].

The present study attempted to address the unavailability of psychometrically robust compassionate health care assessment tools in the Ethiopian context. To fill this gap, the study selected one of the compassionate care of health care provider assessment tools, SCCCS. We have chosen the SCCCS because it is comparatively assessed for its reliability and validation in different settings. This study evaluated the reliability and validity of the SCCCS Amharic version based on a sample of oncology patients. The SCCCS was chosen because it is comparatively has been assessed its reliability and validation in different settings.

## Methods

### Sample size

A single population proportion formula was used to estimate the sample size. The required sample size was determined using EPI info ver.7.2.1.0 stat calc with the proportion of 50%, Z_α/2_ = 1.96, d = 5% and 10% non-response rate, the final sample size was 423.

### Study participants

A total of 423 study participants was selected randomly. The inclusion criteria were age 18 and above years, an oncology patient, no demonstrable sign of confusion, able to provide informed consent, oncology patient who came for a second follow up, admitted oncology patient for a minimum of 2 days, and able to speak Amharic. The participants were asked to rate the degree to which their health care providers made the compassionate care elements apparent during their hospitalization and follow up visit.

### Instrument

The Schwartz Center Compassionate Care Scale^®^ was asked to use (SCCCS) is a 12-item scale that each item is scored on a 10-point scale from 1–10 with response options that range from 1 (not at all successful) to 10 (very successful) [8]. We asked permission to translate and validate the 12-item Schwartz Center Compassionate Care Scale® (SCCCS) in the Ethiopia setting.

### Linguistic validation

The SCCCS tool was translated into Amharic by two people who were fluent in both Amharic and English and was checked by a third person to validate the two person’s translation. After it was made sure that the translators produced the exact Amharic version of the questionnaire, the tool was translated back to English by two other people who were blinded for the original English version of the SCCCS.

### Data collection

The data were collected from March to May 2018. Written informed consent was taken from the study participants. A pre-test was conducted by using a structured Amharic version of SCCCS among 42 oncology patients at the satellite oncology center which is different from the main study site. In the pre-test; study participants were asked about the meaning of each item if the questions were easily understandable if they had the difficulty of understanding the questions and anything they thought that has to be added. The timing was also assessed. The instrument was found to be easily conceivable, simple, clear, and appropriate for the assessment of compassionate care among this group. The time taken to complete the questionnaire ranges from a minimum of ten minutes to a maximum of 20 minutes. And then we proceed to the main study data collection among the inpatients and follow up oncology patients at Tikur Anbessa Specialized Hospital (TASH).

### Data management

The completeness and consistency of each questionnaire were checked. The data were entered into Epi data version 4.2.0.0 and exported to SPSS version 23.0 for cleaning, and analysis.

### Construct validity

#### Confirmatory factor analysis

The appropriateness of the factor structure of the SCCCS identified in previously done studies, which states that the SCCCS is a one-dimensional compassionate care measuring tool [8], was assessed by conducting confirmatory factor analyses using Amos for structural equation modeling.

In the confirmatory factor analysis, different steps were performed. Initially, the normality of the data was assessed through skewness, kurtosis, and, outliers and since the data was normally distributed S1 Table, Maximum likelihood estimation method was selected. There were no missing data. The input matrix was variance-covariance.

The fitness of the model to the sampled data was assessed using several fit indices. Firstly, the Chi^2^ statistic was used as a measure of fit between the sample covariance and fitted covariance matrices. In addition to the Chi^2^ statistic, other fit indices were used to evaluate the fitness of the model such as the Comparative Fit Index (CFI) and the Tucker Lewis Index (TLI). The model with values >0.95 for the CFI and TLI indicates a reasonable fit. The Root Mean Square Error of the Approximation (RMSEA) and Standardized Root Mean Residual (SRMR) where the other fit index that considers assessing the model fitness. RMSEA and SRMR values <0.05 indicate a good model fit [15, 16].

Construct validity of the SCCCS was assessed by convergent validity (factor loadings, average variance extracted and reliability), and discriminant validity. High factor loadings indicate convergence and statistically significant. The average variance extracted (AVE) is calculated as the mean-variance for the items loading on a construct and is a summary of the indicator of convergence. If AVE is greater than 0.5 then we can say that it is adequate convergence. Construct reliability should be > 0.7 to warrant good reliability. The construct validity of the tool was assessed by using the following indicators. These are estimated loadings of 0.5 or higher, AVE of 0.5 or higher to support convergent validity, AVE estimates for two factors should exceed the square of the correlation between two factors to provide evidence of discriminant validity, and, construct reliability should be 0.7 or higher to suggest convergence and internal consistency [15].

The one-factor model is posited whereby the observed measures of show respect (SR), convey information (CI), communicate test results (CT), treat you as a person (TUA), listen to you attentively (LA), Always involve you in decisions about your treatment (AID), gain your trust (GU), considering the effect of your illness (CE), comfortably discuss (CD), express sensitivity, caring, and compassion for your situation (ESC), spend enough time with you (ST), and understand your emotional needs (UE) conjectured to load on a latent dimension of Compassion S1 Fig.

The measurement model presented in S1 Fig, contains 22 freely estimated parameters: 9-factor loadings (SR and CE serve as marker indicators and thus their factor loadings will be fixed), 12 error variances, and 1-factor variances. The model is over-identified with 54*df* (78–24).

The maximum likelihood estimation, overall goodness-of-fit indices suggested that the one-factor structure model does fit poorly the data: X^2^(54) = 450, p<0.001, CMIN/DF = 8.3, SRMR = 0.06, CFI = 0.84, PCFI = 0.69, TLI = 0.80, GFI = 0.83, AGFI = 0.58, PCLOSE = 0.00, and RMSEA = 0.13 (90% CI = 0.12–0.15) S2 Fig.

Using the results of modification when we tried to free the errors but still the one- factor model did not fit well with these data: X^2^(43) = 107.89, p<0.001, CMIN/DF = 2.51, SRMR = 0.04, CFI = 0. .97, PCFI = 0. .63, TLI = 0. 96, GFI = 0.96, AGFI = 0.93, PCLOSE = 0.11, and RMSEA = 0.06 (90% CI = 0.05–0.08) S3 Fig.

The standardized residuals covariance value ranges from 0.000–2.008. This showed the presence of localized areas of ill fit in the solution S2 Table. The standardized factor loadings range from 0.50–0.75. The factor loadings for all items are above 0.5 S3 Table. The value of the average variance extracted (AVE) is 0.43 and the construct reliability is 0.14 which is not acceptable. Based on the above results this one-factor model did not meet the criteria of convergent validity.

As the result of the confirmatory factor analysis showed that the one-factor SCCCS did not fit the data well as per the recommendation of different experts which is to run EFA if the results of the CFA are not satisfactory, we run the exploratory factor analysis [17, 18].

### Structural validity

#### Exploratory factor analysis

Given these overall indicators, factor analysis was deemed to be suitable for all 12 items. Principal axis factoring was used because the primary purpose was to identify the latent factors of the SCCCS. The exploratory factor analysis was done following the next steps. In the first phase, the suitability of the data for factor analysis was checked by running the Kaiser-Meyer-Olkin (KMO) measure of sampling adequacy, Bartlett’s test of sphericity, and the determinant of R matrix. (KMO); shows us whether or not the variables can be grouped into a smaller set of underlying factors. The value of KMO should be 0.5 and above to be considered as suitable for factor analysis. The other thing that we have checked was Bartlett’s test of sphericity which tests the hypothesis that the correlation matrix is an identity matrix; there was no relationship among the item. Bartlett’s Test of Sphericity should be significant (p<0.05) for factor analysis to be suitable. To avoid extreme multicollinearity and singularity; which shows perfect correlation, determinant of R matrix; in which its value should be >0.00001 have been also assessed.

In the second phase, we have undergone the factor extraction analysis using the principal axis factoring (PAF) way of factor extraction. The eigenvalue, scree test, and cumulative percentages of variance were used as criteria to determine the number of common factors to be retained. Factors with eigenvalues of greater than 1.0 were kept and the rest was discarded. Together with the PAF, a Varimax rotation method was selected to have a more interpretable and simplified solution. Items were removed from the EFA if they are double-loaded (i.e., 0.40 or above on more than one factor), unique, and do not load into any factor [19, 20]. The reliability of the factor structure got from the EFA was assessed by running Cronbach’s alpha. Reliability greater than 0.8 is considered as high [21].

### Ethical consideration

All study participants were informed about the objectives of the study and written informed consent was obtained from each participant before the interview. Participation in the study was voluntary. The study was approved by the Institutional Review Board (IRB) of the College of Health Sciences at Addis Ababa University.

## Results

### Sample characteristics

During the data collection period, 9 study participants refused. This resulted in a final sample size of 414 participants with a 97.9% response rate.

Among the study participants, 275 (66.4%) were females. The mean age was 47 years, SD = ±13.05, range = 18 to 75. Martially, 288 (69.6%) of the participants were married, and only 125 (30.2%) participants were educated up to secondary school. Breast cancer was found to be the leading cause of facility visit 122(29.5%) followed by cervical cancer 83(20%) Table 1.

**Table 1:** Sociodemographic characteristics of oncology patients at TASH, Addis Ababa, Ethiopia, 2018 (n = 414).

Characteristics	Frequency (%)	Characteristics	Frequency (%)
**Sex**		**Disease category**	
Male	139 (33.6)	Breast cancer	122 (29.5)
Female	275 (66.4)	CUP	2 (0.5)
**Mean age** ±**SD**	47±13.05	Cervical cancer	83 (20)
**Religion**		Ano-rectal cancer	43 (10.4)
Orthodox	306 (73.9)	Esophageal cancer	8 (1.9)
Muslim	66 (15.9)	Gastric cancer	8 (1.9)
Protestant	37 (8.9)	Kaposi’s sarcoma	1 (0.2)
Catholic	3 (0.7)	Liver ca (HCC)	7 (1.7)
Other	2 (0.5)	Lung cancer	6 (1.4)
**Marital status**		NPC	8 (1.9)
Single	62 (15.0)	NHL	6 (1.4)
Married	288 (69.6)	Ovarian cancer	5 (1.2)
Divorced	15 (3.6)	Prostatic cancer	6 (1.4)
Separated	9 (2.2)	Renal cell cancer	2 (0.5)
Widowed	40 (9.7)	Sarcoma	10 (2.4)
**Educational status**		Testicular cancer	8 (1.9)
Cannot read and write	83 (20)	Thyroid cancer	26 (6.3)
Can read and write	21 (5.1)	Uterine Fibroma	3 (0.7)
Primary (grade 1–8)	78 (18.8)	Vulvar cancer	1 (0.2)
Secondary (9–12)	125 (30.2)	Other Neoplasms	59 (14.3)
College and above	107 (25.8)		
**Contacting the patient**			
At inpatient ward	78 (18.8)		
At follow up clinic	336 (81.2)		

CUP, Cancer of Unknown Primary Origin; HCC, Hepatocellular Cancer; NPC, Naso-pharyngeal Cancer; NHL, Non-Hodgkin’s Lymphoma

### Factor analysis

For this study’s sample, the KMO measure of adequate sampling was 0.906. This result indicates that the data represented a homogeneous collection of variables that were suitable for factor analysis. Meanwhile, Bartlett’s test of sphericity was also significant for the sample [x ^2^ = 2499.825, df = 66, p < .001)], which indicates that the set of correlations in the correlation matrix Table 2 were significantly different from zero and thus suitable for factor analysis. The determinant of the R matrix was 0.002. Further confirming that each item shared some common variance with other items.

**Table 2:** Correlation matrix for the 12 SCCCS items (n = 414).

Correlation Matrix											
		SR	CI	CT	TUA	LA	GU	CE	CD	ST	UE
Correlation	SR	1.00	0.51	0.40	0.65	0.50	0.40	0.36	0.44	0.34	0.43
	CI	0.51	1.00	0.37	0.61	0.57	0.36	0.43	0.47	0.47	0.49
	CT	0.40	0.37	1.00	0.36	0.33	0.38	0.25	0.24	0.39	0.28
	TUA	0.65	0.61	0.36	1.00	0.68	0.40	0.38	0.42	0.42	0.42
	LA	0.50	0.57	0.33	0.68	1.00	0.39	0.39	0.49	0.43	0.50
	GU	0.40	0.36	0.38	0.40	0.39	1.00	0.37	0.39	0.53	0.36
	CE	0.36	0.43	0.25	0.38	0.39	0.37	1.00	0.60	0.47	0.60
	CD	0.44	0.47	0.24	0.42	0.49	0.39	0.60	1.00	0.45	0.70
	ST	0.34	0.47	0.39	0.42	0.43	0.53	0.47	0.45	1.00	0.60
	UE	0.43	0.49	0.28	0.42	0.50	0.36	0.56	0.70	0.60	1.00

In the Exploratory factor analysis, of the 12 items, 10 items had high loading regarding their intended factors, while two items (i.e., item 6 and item 10) have double-loaded and the items were dropped Table 3.

**Table 3:** Factor loadings based on a principal axis factoring with Varimax rotation for 12 items of the SCCCS (n = 414).

The 12 items of the Schwartz center compassionate care scale	EFA		Item in the factors
	Component		
	First factor	Second factor	
Show respect for you, your family, and those important to you	0.65		Retained in Factor 1
Convey information to you in an understandable way	0.63		Retained in Factor 1
Communicate test results in a timely and sensitive manner	0.46		Retained in Factor 1
Treat you as a person, not just a disease	0.82		Retained in Factor 1
Listen to you attentively	0.67		Retained in Factor 1
Always involve you in decisions about your treatment	0.49	0.41	**Dropped**
Gain your trust	0.44		Retained in Factor 1
Consider the effect of your illness on you, your family, and the people most important to you		0.61	Retained in Factor 2
Comfortably discuss sensitive, emotional, or psychological issues		0.73	Retained in Factor 2
Express sensitivity, caring, and compassion for your situation	0.46	0.60	**Dropped**
Spend enough time with you		0.57	Retained in Factor 2
Strive to understand your emotional needs		0.84	Retained in Factor 2

Furthermore, the analysis was conducted for the remaining 10 items. The KMO measure of adequate sampling, Bartlett’s test of sphericity, and the determinant of R matrix were done for a second time to assess the suitability of the data for factor analysis on the remaining 10 items. The KMO measure of adequate sampling was determined to be 0.88. Bartlett’s test of sphericity was significant for the sample [x ^2^ = 1940.835 df = 45, p < .000)], which indicates that the set of correlations in the correlation matrix were significantly different from zero and thus it is suitable for factor analysis and the determinant of R matrix value was 0.009. The result yielded a two-factor structure, with factor loadings of items that settled at each subscale of the SCCCS with two factors in which their loading varied between 0.42 and 0.85 Table 4. Factor 1 explained 27.114% of the total variance (eigenvalue = 5.031); factor 2 (explained 52.616% (eigenvalue = 1.073) S4 Table and S4 Fig. The communalities were also above 0.3 except for item 3(communicate test results timely) Table 4.

**Table 4:** Factor loadings and communalities based on a principal axis factoring with Varimax rotation for 10 items of the SCCCS (n = 414).

The 12 items of the Schwartz center compassionate care scale	EFA		Item in the factors	Communality
	Component			
	First factor	Second factor		
Show respect for you, your family, and those important to you	0.66		Retained in Factor 1	0.50
Convey information to you in an understandable way	0.62		Retained in Factor 1	0.55
Communicate test results in a timely and sensitive manner	0.44		Retained in Factor 1	0.26
Treat you as a person, not just a disease	0.85		Retained in Factor 1	0.72
Listen to you attentively	0.65		Retained in Factor 1	0.59
Gain your trust	0.42		Retained in Factor 1	0.33
Consider the effect of your illness on you, your family, and the people most important to you		0.46	Retained in Factor 2	0.45
Comfortably discuss sensitive, emotional, or psychological issues		0.73	Retained in Factor 2	0.62
Spend enough time with you		0.59	Retained in Factor 2	0.48
Strive to understand your emotional needs		0.79	Retained in Factor 2	0.77

This two-factor structure tool was given name, in which the first latent factor which contains show respect (SR), convey information (CI), communicate test results (CT), treat you as a person (TUA), listen to you attentively (LA), and gain your trust (GU) items as recognizing suffering, and the second latent factor which contains considering the effect of your illness (CE), comfortably discuss (CD), spend enough time with you (ST), and understand your emotional need (UE) items as acting to relieve suffering.

Internal consistency for all items in one and each of the scales was examined using Cronbach’s alpha. The overall reliability was 0.88, and 0.84 for both recognizing suffering (6 items), and acting to relieve suffering (4 items) Table 5.

**Table 5:** Descriptive statistics for the two factor SCCCS (n = 414).

S.N	Factor name	No. of items	Mean(SD)	Skewness	Kurtosis	Cronbach’s alpha
1	SCCCS	10				0.88
2	Recognizing suffering	6	8.7 (0.50)	-0.15	-1.99	0.84
3	Acting to relieve suffering	4	8.0 (0.50)	0.19	-1.98	0.84

Composite scores were created for the two factors, based on the mean of the items which had their primary loadings on each factor. The skewness and kurtosis were well within a tolerable range for assuming a normal distribution Table 5.

## Discussion

Overall, this study indicated that two factors were underlying SCCCS items, based on the principal axis factoring with Varimax rotation, and these factors were highly internally consistent. Two of the twelve items were eliminated, and the original factor structure proposed by Beth A. Lown, SJM, Raymond, and Chadwick (2015) was not retained [8].

The validity analyses provided psychometric support that the SCCCS could be used in a local context with two dimensions and 10 items to better measure compassionate care practice among oncology patients. The results also showed that the scale has high internal consistency. The exploratory factor analysis finding of the current study is contradicted with the finding of a study done in the USA, which showed that the SCCCS is a one-dimensional factor structure compassionate care measuring tool [8]. The possible reason for the observed difference could be due to the analysis procedure followed; in the USA study in which firstly the 16 items were split into two item sets and administered to 800 recently hospitalized patients and 510 physicians; half were asked to answer item set one and a half item set two. The authors conducted EFA for each set of items separately and concluded that items within each set were one-dimensional. However, they did not conduct analyses on all the items, making it impossible to determine whether the scale as a whole is one-dimensional, or whether the measure consisted of two separate scales or subscales.

Another validity and reliability study on the SCCCS was done in Ireland and the result showed that the SCCCS is one-dimensional with 12 items compassionate care practice measurement tool [12], which is still against the current study finding. The reason for this disparity could be due to technical differences in the study participant selection, data collection, and analysis path followed in studies conducted in the USA and Ireland. The other possible reason could be the socio-cultural difference.

### Limitations

The finding of this study contributed that a contextually validated tool other than where it is developed will help in becoming a tool in studies that assess compassionate care of health care providers but the following limitations should be taken into account. The applicability of this tool could be only among oncology patients that it might not have similar findings among other non-oncology and acutely ill patients. The other limitation is that this study assessed the reliability by calculating Cronbach’s alpha that the test-retest reliability which shows the stability of the finding overtime was not done.

## Conclusions

In this study, we found that the Schwartz Center Compassionate Care Scale has high internal consistency and acceptable structural validity value among oncology patients. By considering the validity, it is reasonable to conclude that the Schwartz Center Compassionate Care Scale can be applied in measuring compassionate care practice in the Ethiopian setting.

## Supporting information

S1 Fig Path diagram and input data for the one-factor CFA model of SCCCS. SR = show respect, CI = convey information, CT = communicate test results, TUA = treat you as a person, LA = listen to you attentively, AID = Always involve you in decisions about your treatment, GU = gain your trust, CE = considering the effect of your illness, CD = comfortably discuss, ESC = express sensitivity, caring, and compassion for your situation, ST = spend enough time with you, and UE = understand your emotional needs.
(DOCX)

S2 Fig One factor model of the SCCCS with standardized estimates. (DOCX)

S3 Fig One factor model of the SCCCS with standardized estimates and co-variance between errors. (DOCX)

S4 Fig Scree plot for the for the 12 SCCCS items (n = 414). (DOCX)

S1 Table Assessment of normality for the 12 SCCCS items (n = 414). (DOCX)

S2 Table Standardized residual covariances for the 12 SCCCS items (n = 414). (DOCX)

S3 Table Standardized regression weights for the 12 SCCCS items (n = 414). (DOCX)

S4 Table Total variance explained result with principal axis factoring for the 12 SCCCS items (n = 414). (DOCX)

S1 Questionnaire (DOCX)

S2 Questionnaire (DOCX)

## References

[1] Lown B, Julie R, John M. An Agenda For Improving Compassionate Care: A Survey Shows About Half Of Patients Say Such Care Is Missing. Health Affairs. 2011;30(9):1772–8. 10.1377/hlthaff.2011.0539 21900669

[2] Lown B. Seven guiding commitments: Making the U.S. healthcare system more compassionate. Journal of Patient Experience. 2014;1(2). 10.1177/237437431400100203 PMC5513594 28725803

[3] Blewitt L, Karyn Wang, Nguyen H, Johnson A, Pidial K, Yu N. Mindfulness: Creating the Space for Compassionate Care. 2015.

[4] Shea S, Lionis C. Introducing the Journal of Compassionate Health Care. Compassionate Health Care. 2014;1(7).

[5] Epstein RM, Franks P, Shields CG, Meldrum SC, Miller KN, Campbell TL, et al. Patient-Centered Communication and Diagnostic Testing. Annals of Family Medicine. 2005;3:415–21. 10.1370/afm.348 16189057 PMC1466928

[6] Donald J C, Richard L SJ, Clinch CR. The Impact of Patient Participation on Physicians’ Information Provision During a Primary CareMedical Interview. Health Communication. 2007;21(2):177–85. 10.1080/10410230701307824 17523863

[7] Fogarty L, Curbow B, Wingard J, McDonnell K, Somerfield M. Can 40 Seconds of Compassion Reduce Patient Anxiety. Clinical Oncology. 1999;17(1). 10.1200/JCO.1999.17.1.371 10458256

[8] Lown BA, Muncer SJ, Chadwick R. Can compassionate healthcare be measured? The Schwartz Center Compassionate Care ScaleTM, Patient Educ Couns. 2015; 98: 1005–1010. 10.1016/j.pec.2015.03.019 25921380

[9] Burnell L, L. Agan D. Compassionate Care: Can it be Defined and Measured? The Development of the Compassionate Care Assessment Tool. International Journal of Caring Sciences. 2013;6(2).

[10] Sinclair S, Jaggi P, Hack T, McClement S, Bouchal S, Singh P. Assessing the credibility and transferability of the patient compassion model in non- cancer palliative populations. BMC Palliative Care. 2018;17(108). 10.1186/s12904-018-0358-5 30213263 PMC6137734

[11] Strauss C, Taylor B, Gu J, Kuyken W, Baer R, Jones F, et al. What is compassion and how can we measure it? A review of definitions and measures. Clinical Psychology Review. 2016;47:15–27. 10.1016/j.cpr.2016.05.004 27267346

[12] Lown B, Dunne H, Muncer S, Chadwick R. How important is compassionate healthcare to you? A comparison of the perceptions of people in the United States and Ireland. Research in Nursing. 2017;22(1–2):60–9.

[13] Manning C. Predictors of Compassionate Care. 2016.

[14] Sinclair S, Russell L, Hack T, Kondejewski J, Sawatzky R. Measuring Compassion in Healthcare: A Comprehensive and Critical Review. Springer. 2016. 10.1007/s40271-016-0209-5 27866323

[15] Brown TA. Confirmatory Factor Analysis for Applied Research. Kenny DA, editor. New York: The Guilford Press; 2006.

[16] Mulaik SA. Foundations of Factor Analysis. Cameron AC, Long JS, Gelman A, Rabe-Hesketh S, Skrondal A, editors. USA: Taylor & Francis Group, LLC; 2010.

[17] Karin SE. Is it compulsory to do confirmatory factor analysis? 2015 [Available from: https://www.researchgate.net/post/Is_it_compulsory_to_do_confirmatory_factor_analysis_when_I_am_going_to_translate_a_questionnaire/55bf29575cd9e36f108b45cd/citation/download.

[18] Hurley AE, Et al. Exploratory and confirmatory factor analysis: guidelines, issues, and alternatives. Journal of Organizational Behavior. 1997;18:667–83.

[19] Williams B, Onsman A, Brown T. Exploratory factor analysis: A five-step guide for novices. JEPHC. 2010;8(3).

[20] Taherdoost H, Sahibuddin S, Jalaliyoon N. Exploratory Factor Analysis; Concepts and Theory. Advances in Applied and Pure Mathematics.

[21] Mohajan H. Two Criteria for Good Measurements in Research: Validity and Reliability. Annals of Spiru Haret University. 2017;17(3):59–82.

