# Supplementary material for: Validity and reliability of the Amharic version of the Schwartz Center Compassionate Care Scale
Source: PLoS One. 2021 Mar 23;16(3):e0248848. doi: 10.1371/journal.pone.0248848 (PMC7987159; doi:10.1371/journal.pone.0248848)
Supplement: S1 Fig — SR = show respect, CI = convey information, CT = communicate test results, TUA = treat you as a person, LA = listen to you attentively, AID = Always involve you in decisions about your treatment, GU = gain your trust, CE = considering the effect of your illness, CD = comfortably discuss, ESC = express sensitivity, caring, and compassion for your situation, ST = spend enough time with you, and UE = understand your emotional needs. (DOCX) [file pone.0248848.s001.docx]

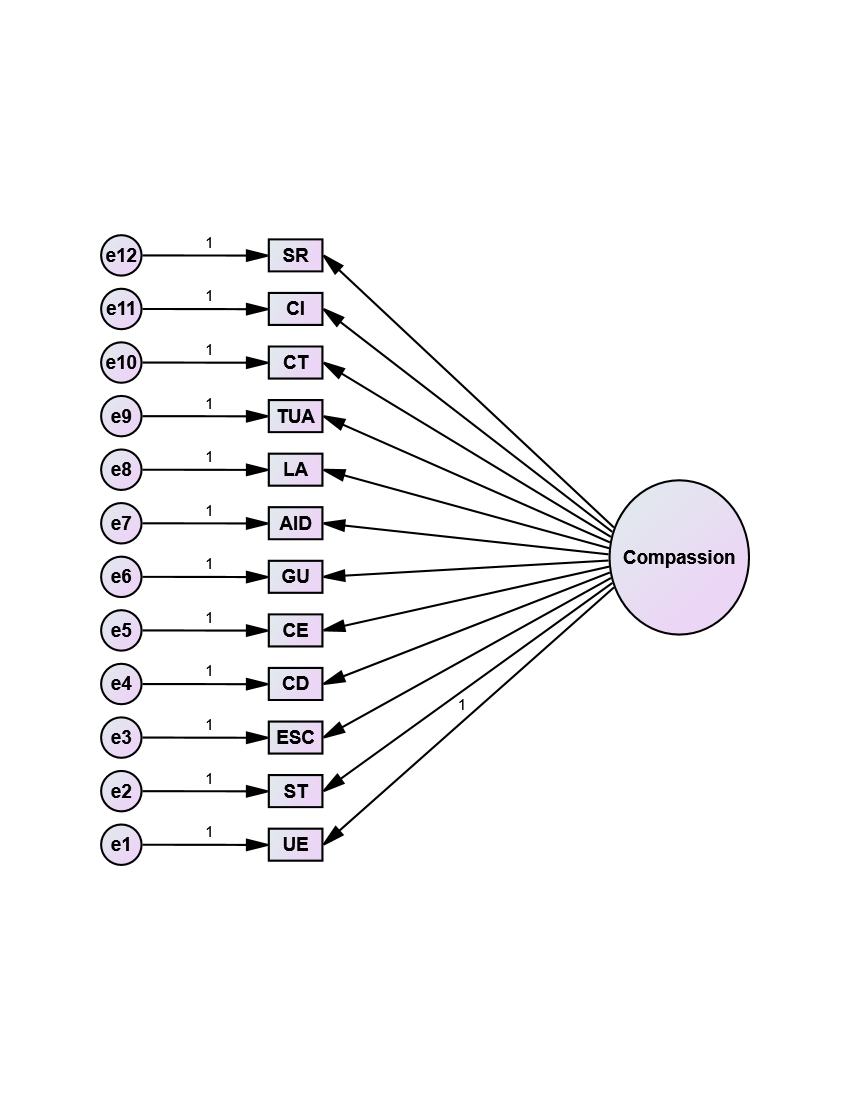


**S1 Fig. Path diagram and input data for the one-factor CFA model of SCCCS.**

**SR=show respect, CI=convey information, CT=communicate test results, TUA=treat you as a person, LA=listen to you attentively, AID=Always involve you in decisions about your treatment, GU=gain your trust, CE=considering the effect of your illness, CD=comfortably discuss, ESC=express sensitivity, caring, and compassion for your situation, ST=spend enough time with you, and UE=understand your emotional needs.**
