# Supplementary figures and images for: Validity and reliability of the Amharic version of the Schwartz Center Compassionate Care Scale
Source: PLoS One. 2021 Mar 23;16(3):e0248848. doi: 10.1371/journal.pone.0248848 (PMC7987159; doi:10.1371/journal.pone.0248848)

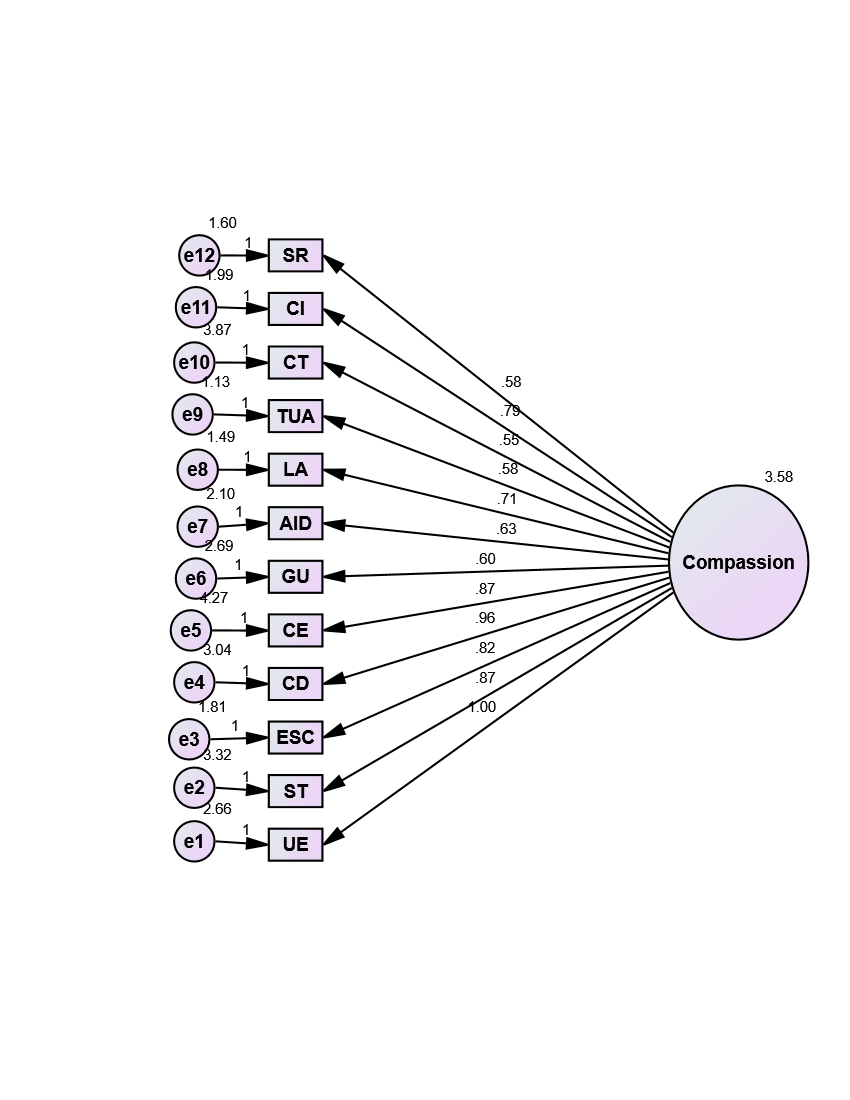


S2 Fig. A one factor model of the SCCCS with standardized estimates.

Supplement: S2 Fig — (DOCX) [file pone.0248848.s002.docx]

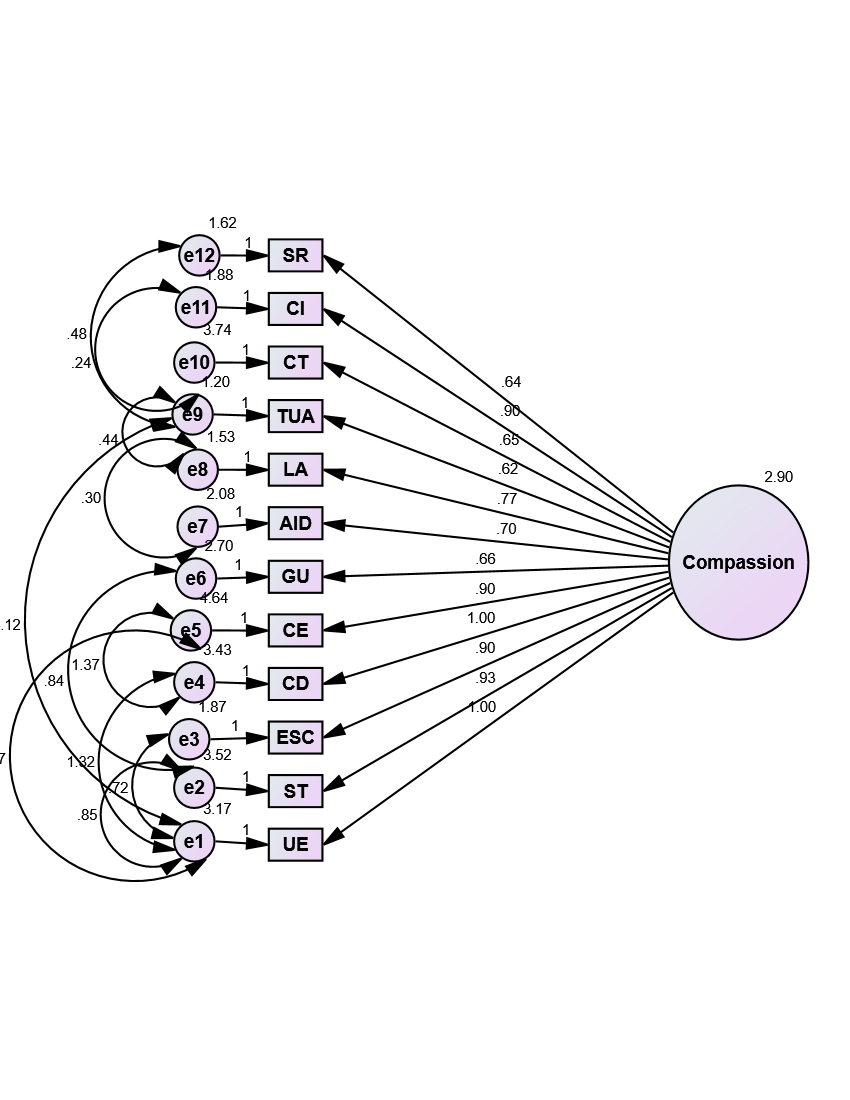


S3 Fig. One factor model of the SCCCS with standardized estimates and co-variance between errors.

Supplement: S3 Fig — (DOCX) [file pone.0248848.s003.docx]

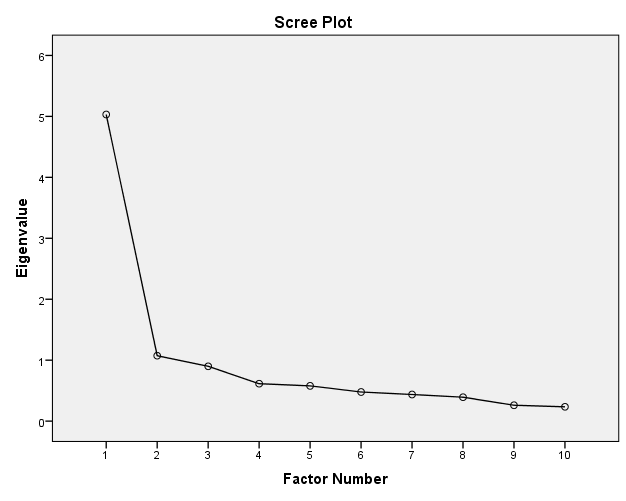


**S4 Fig. Scree plot for the for the 12 SCCCS items (n=414)**

Supplement: S4 Fig — (DOCX) [file pone.0248848.s004.docx]
