# Supplementary material for: Validity and reliability of the Amharic version of the Schwartz Center Compassionate Care Scale
Source: PLoS One. 2021 Mar 23;16(3):e0248848. doi: 10.1371/journal.pone.0248848 (PMC7987159; doi:10.1371/journal.pone.0248848)
Supplement: S1 Table — (DOCX) [file pone.0248848.s005.docx]

##### **S1 Table: Assessment of normality for the 12 SCCCS items (n=414)**

| **Variable** | **min** | **max** | **Skew** | **c.r.** | **kurtosis** | **c.r.** |
| --- | --- | --- | --- | --- | --- | --- |
| PR1 | 1.000 | 10.000 | -1.902 | -15.803 | 3.776 | 15.685 |
| PI2 | 1.000 | 10.000 | -1.705 | -14.160 | 2.891 | 12.009 |
| PC3 | 1.000 | 10.000 | -1.383 | -11.489 | 1.397 | 5.801 |
| PT4 | 1.000 | 10.000 | -2.414 | -20.051 | 7.173 | 29.792 |
| PL5 | 1.000 | 10.000 | -2.249 | -18.682 | 5.601 | 23.262 |
| PI6 | 1.000 | 10.000 | -2.040 | -16.942 | 4.804 | 19.954 |
| PG7 | 1.000 | 10.000 | -1.737 | -14.426 | 2.848 | 11.829 |
| PE8 | 1.000 | 10.000 | -1.427 | -11.855 | 1.145 | 4.757 |
| PD9 | 1.000 | 10.000 | -1.601 | -13.302 | 1.823 | 7.573 |
| PS10 | 1.000 | 10.000 | -2.032 | -16.876 | 4.093 | 16.999 |
| PTS11 | 1.000 | 10.000 | -1.281 | -10.643 | .892 | 3.704 |
| PEN12 | 1.000 | 10.000 | -1.559 | -12.954 | 1.630 | 6.770 |
| Multivariate |  |  |  |  | 228.168 | 126.635 |
