# Supplementary material for: Validity and reliability of the Amharic version of the Schwartz Center Compassionate Care Scale
Source: PLoS One. 2021 Mar 23;16(3):e0248848. doi: 10.1371/journal.pone.0248848 (PMC7987159; doi:10.1371/journal.pone.0248848)
Supplement: S2 Table — (DOCX) [file pone.0248848.s006.docx]

**S2 Table. Standardized Residual Covariances for the 12 SCCCS items (n=414)**

|  | PR1 | PI2 | PC3 | PT4 | PL5 | PI6 | PG7 | PE8 | PD9 | PS10 | PTS11 | PEN12 |
| --- | --- | --- | --- | --- | --- | --- | --- | --- | --- | --- | --- | --- |
| PR1 | .000 |  |  |  |  |  |  |  |  |  |  |  |
| PI2 | .634 | .000 |  |  |  |  |  |  |  |  |  |  |
| PC3 | 1.785 | .595 | .000 |  |  |  |  |  |  |  |  |  |
| PT4 | 3.310 | 1.566 | .477 | .000 |  |  |  |  |  |  |  |  |
| PL5 | .278 | .614 | -.387 | 2.599 | .000 |  |  |  |  |  |  |  |
| PI6 | -.938 | .371 | .840 | -.286 | 1.638 | .000 |  |  |  |  |  |  |
| PG7 | .607 | -1.056 | 2.266 | -.097 | -.534 | 1.274 | .000 |  |  |  |  |  |
| PE8 | -.876 | -.506 | -.836 | -1.207 | -1.313 | .031 | .290 | .000 |  |  |  |  |
| PD9 | -.519 | -.998 | -1.965 | -1.743 | -.774 | -.155 | -.401 | 2.747 | .000 |  |  |  |
| PS10 | -.514 | .257 | -.575 | -.552 | -.254 | -.723 | -.805 | -1.136 | .417 | .000 |  |  |
| PTS11 | -1.877 | -.265 | 1.459 | -1.233 | -1.132 | -.261 | 2.839 | .909 | -.609 | .208 | .000 |  |
| PEN12 | -1.160 | -1.152 | -1.517 | -2.226 | -1.061 | -.834 | -1.329 | 1.521 | 2.700 | 1.882 | 1.673 | .000 |
