# Supplementary material for: Validity and reliability of the Amharic version of the Schwartz Center Compassionate Care Scale
Source: PLoS One. 2021 Mar 23;16(3):e0248848. doi: 10.1371/journal.pone.0248848 (PMC7987159; doi:10.1371/journal.pone.0248848)
Supplement: S3 Table — (DOCX) [file pone.0248848.s007.docx]

| Items | Estimate |
| --- | --- |
| UE | 0.69 |
| ST | 0.65 |
| ESC | 0.75 |
| CD | 0.68 |
| CE | 0.58 |
| GU | 0.56 |
| AID | 0.64 |
| LA | 0.73 |
| TUA | 0.70 |
| CT | 0.50 |
| CI | 0.74 |
| SR | 0.65 |

**S3 Table. Standardized regression weights for the 12 SCCCS items (n=414)**
