# Supplementary material for: Validity and reliability of the Amharic version of the Schwartz Center Compassionate Care Scale
Source: PLoS One. 2021 Mar 23;16(3):e0248848. doi: 10.1371/journal.pone.0248848 (PMC7987159; doi:10.1371/journal.pone.0248848)
Supplement: S4 Table — (DOCX) [file pone.0248848.s008.docx]

**S4 Table: Total Variance Explained result with principal axis factoring for the 12 SCCCS items (n=414)**

| **Total Variance Explained** | | | | | | | | | |
| --- | --- | --- | --- | --- | --- | --- | --- | --- | --- |
| Factor | Initial Eigenvalues | | | Extraction Sums of Squared Loadings | | | Rotation Sums of Squared Loadings | | |
|  | Total | % of Variance | Cumulative % | Total | % of Variance | Cumulative % | Total | % of Variance | Cumulative % |
| 1 | 5.03 | 50.31 | 50.31 | 4.59 | 45.86 | 45.86 | 2.71 | 27.11 | 27.11 |
| 2 | 1.07 | 10.73 | 61.05 | 0.68 | 6.76 | 52.62 | 2.55 | 25.50 | 52.62 |
| 3 | 0.90 | 8.00 | 70.04 |  |  |  |  |  |  |
| 4 | 0.61 | 6.14 | 76.18 |  |  |  |  |  |  |
| 5 | 0.58 | 5.78 | 81.97 |  |  |  |  |  |  |
| 6 | 0.48 | 4.78 | 86.75 |  |  |  |  |  |  |
| 7 | 0.44 | 4.37 | 91.12 |  |  |  |  |  |  |
| 8 | 0.39 | 3.92 | 95.04 |  |  |  |  |  |  |
| 9 | 0.26 | 2.61 | 97.65 |  |  |  |  |  |  |
| 10 | 0.24 | 2.35 | 100.00 |  |  |  |  |  |  |
