# Supplementary material for: Validity and reliability of the Amharic version of the Schwartz Center Compassionate Care Scale
Source: PLoS One. 2021 Mar 23;16(3):e0248848. doi: 10.1371/journal.pone.0248848 (PMC7987159; doi:10.1371/journal.pone.0248848)
Supplement: S1 Questionnaire — (DOCX) [file pone.0248848.s009.docx]

# **S1 Questionnaire. English version questionnaire**

1. **Study Information Sheet**

Good day: My name is ………………………………….. I am here on behalf of Merkeb Zeray, a student of Addis Ababa University School of public health. She is conducting a research on “Validity and reliability of the Schwartz Center Compassionate Care Scale tool Amharic Version in the Ethiopian context". She received permission from Addis Ababa university school of public health and clinical oncology unit of Tikur Anbessa Hospital to conduct this study.

You are selected by systematic random sampling method to participate in this study because you are currently attending inpatient care/ follow-up. Your participation is purely based on your willingness. You have the right to choose not to take part in this study. If you choose to take part, you have the right to stop at any time. If you are willing to participate or refuse or decide to withdraw later, you will not be subjected to any ill-treatment.

If you agree to participate in the study, you will be asked to answer some questions about yourself, compassionate care practice of health care provider.

The study will provide baseline data for policymakers and other researchers for further improvements of compassionate care. Your responses will be kept confidential and names will not be written in the questionnaire and only codes will be used and this data will not be used for any purposes other than this study. Participating in this study will not have any kind of risks and you may not directly benefit or get payment from participating in this study. Completing this questionnaire can take about 10 to 20 minutes of your time and we greatly appreciate your cooperation. If you have any question which is not clear for you, you welcome to ask at any time.

If you need any further information or explanation regarding to this study, you can contact the principal investigator. Here are her contact details;

Address: Cell phone   +251967036236

Email: [merkebzeray@gmail.com](mailto:merkebzeray@gmail.com)

Do I have your permission to continue?

Yes No

If yes, thank him/her and proceed to the informed consent and to the questionnaire

If no, thank him/her and go to the next participant

**B. Written Consent Form**

I have understood this form and clearly understood its purpose, what is expected from me and I am willing to voluntarily participate in this study.

Informed consent Certified by:

Respondent’s signature ____________________________Date______________________

Data collector: Name______________________________ Signature_________________

Date of interview (Ethiopian calendar)____/__/___________

Time started____________

Time completed___________

Result of interview:

1. Completed………………

2. Refused …………….

3. Respondent not available…………..

4. Partially completed …

Checked by supervisor; Name ………………………….. Signature ………………… Date ………………...

**Part I: Characteristics of patient respondents**

**Instruction: For each of the following questions please circle the alternative that fit for respondent’s response.**

| Code | Question | Answer category | Skip |
| --- | --- | --- | --- |
| 101 | Sex | 1. Male 2. Female |  |
| 102 | What is your age in completed years? | _____________________ |  |
| 103 | The place(region) he/she comes from | 1. Tigray 2. Afar 3. Amhara 4. Oromo 5. Somali 6. Benshangul Gumuz 7. Southern nations and nationality people 8. Gambella 9. Harari 10. Addis Ababa 11. DireDawa |  |
| 104 | Residence | 1. Urban 2. Rural |  |
| 105 | Ethnicity | __________________________ |  |
| 106 | What is your religion? | 1. Orthodox 2. Muslim 3. Protestant 4. Catholic 5. Other specify___ |  |
| 107 | What is your current marital status? | 1. Single 2. Married 3. Divorced 4. Separated 5. Widowed |  |
| 108 | What is your educational level? | 1. Cannot read and write 2. Can read and write 3. Grade 1-6 4. Grade 7-12 5. Diploma and above |  |

**Part II: Patient’s medical history**

**Instruction: This part of questionnaire deals with study participant’s information about their illness, hospitalization and frequency of visit. Please ask the study participants the provided questions and with great attention to the skip pattern.**

| Code | Question | Answer category | Skip |
| --- | --- | --- | --- |
| 201 | Where does the patient found | 1. At inpatient oncology ward 2. At oncology OPD | If the study participant is an outpatient then skip to Q206 |
| 202 | Full name of the diagnosis |  | To be filled from patient card |
| 203 | Number of hospitalization days(inpatients) | ___________ |  |
| 204 | How many times have you hospitalized including your current admission since you started the treatment? | _____________________ | Go to Q301 |
| 205 | How many visits have you made including your today visit since you started the treatment? | _____________________________ | Skip to Q301 for inpatient study participant. |
| 206 | Have you ever been hospitalized at Tikur Anbessa Hospital for this case? | 1. Yes 2.No | If no skip to Q301 |
| 207 | How many days were you hospitalized during your last admission? | _____________________ |  |

**Part III: The Schwartz Center Compassionate Care Scale tool**

**Instructions:** **For the following questions please mark on 1 when the patient says the doctor (or other healthcare provider) not at all successful, mark on 10 when the patient says the doctor (or other healthcare provider is very successful and for neutral mark on 2,3,4,5,6,7,8 and 9.**

| **Code** | **Elements of compassionate care** | **1** | **2** | **3** | **4** | **5** | **6** | **7** | **8** | **9** | **10** | **Skip** |
| --- | --- | --- | --- | --- | --- | --- | --- | --- | --- | --- | --- | --- |
| “Now, I would like to turn to an approach to treating patients known as compassionate health care that focuses on improving the relationships between doctors, nurses and other professional caregivers and patients and their families. Its particular focus is to improve the communication and emotional support that patients receive from their doctors, nurses and other professional caregivers.  On a scale of 1 to 10, where 1 is not at all successful and 10 is very successful, how successfully did your doctor (or other healthcare provider): | | | | | | | | | | | | |
| **301** | Show respect for you, your family, and those important to you |  |  |  |  |  |  |  |  |  |  |  |
| **302** | Convey information to you in a way that is understandable |  |  |  |  |  |  |  |  |  |  |  |
| **303** | Communicate test results in a timely and sensitive manner |  |  |  |  |  |  |  |  |  |  |  |
| **304** | Treat you as a person, not just a disease |  |  |  |  |  |  |  |  |  |  |  |
| **305** | Listen attentively to you |  |  |  |  |  |  |  |  |  |  |  |
| **306** | Always involve you in decisions about your treatment |  |  |  |  |  |  |  |  |  |  |  |
| **307** | Gain your trust |  |  |  |  |  |  |  |  |  |  |  |
| **308** | Consider the effect of your illness on you, your family, and the people most important to you |  |  |  |  |  |  |  |  |  |  |  |
| **309** | Comfortably discuss sensitive, emotional, or psychological issues |  |  |  |  |  |  |  |  |  |  |  |
| **310** | Express sensitivity, caring, and compassion for your situation |  |  |  |  |  |  |  |  |  |  |  |
| **311** | Spend enough time with you |  |  |  |  |  |  |  |  |  |  |  |
| **312** | Strive to understand your emotional needs |  |  |  |  |  |  |  |  |  |  |  |

**Thank You!**
