# Supplementary material for: Validity and reliability of the Amharic version of the Schwartz Center Compassionate Care Scale
Source: PLoS One. 2021 Mar 23;16(3):e0248848. doi: 10.1371/journal.pone.0248848 (PMC7987159; doi:10.1371/journal.pone.0248848)
Supplement: S2 Questionnaire — (DOCX) [file pone.0248848.s010.docx]

# S2 Questionnaire. Amharic version questionnaire

**አዲስ አባባ ዩኒቨርሲቲ**

**ጤና ሳይንስ ኮሌጅ**

**የማህበረ-ሰብ ጤና ትምህርት ቤት**

ሀ. የስምምነት ቅፅ

እንደምንአደረችሁ/ዋላችሁ፣ስሜ______________ይባላል፡፡እዚህ የመጣሁት በአዲስ አባባ ዩኒቨርሲቲ ጤና ሳይንስ ኮሌጅ የማህበረ-ሰብ ጤና ትምህርት ቤት ተማሪ የሆኑት መርከብ ዘርኣይ በጥቁር ኣንበሳ ሆስፒታል በክትትል እና ተኝተው በመታከም ላይ ያሉ ካንሰር ህመምተኞች ርህራሄ ያማከለ የጤና ባለሙያዎች ህክምና ኣሰጣጥ ዙርያ ለሚያደርጉት ጥናት መረጃ ለመሰብሰብ ነው። ይህንን ጥናት እንዲያካሂዱም ከአዲስ አባባ ዩኒቨርሲቲ ጤና ሳይንስ ኮሌጅ የማህበረ-ሰብ ጤና ትምህርት ቤት እና ጥቁር ኣንበሳ ሆስፒታል ካንሰር ህክምና ክፍል ፍቃድ ኣግኝተዋል።

በዚህ ጥናት እንድትሳተፉ የተመረጣችሁ በአጋጣሚና በፈላጎታችሁ ነው፡፡. በዚህ ጥናት የመሳተፍና ያለመሳተፍ እንዲሁም ለመመለስ የማትፈልጉት ጥያቄዎች ካሉ ያለመመለስና በማንኛውም ጊዜ በጥናቱ ላይ ላለመሳተፍ መወሰን ይችላሉ፡፡ ይሁን እንጂ ለእነዚህ ጥያቄዎች የሚሰጡት ታማኝ መልሶች በጥቁር ኣንበሳ ሆስፒታል በክትትል እና ተኝተው በመታከም ላይ ያሉ ካንሰር ህመምተኞች ርህራሄን ያማከለ የጤና ባለሙያዎች ህክምና ኣሰጣጥ ዙርያ ምን እንደሚመስል ለማወቅ ይረዱናል፡፡ የሚሰጡን መረጃ ሚስጥራዊነቱ የተጠበቀ እና ለጥናታዊ ተግባር ብቻ የሚዉል እና ለማንም የማይገለጽ ይሆናል፡፡ ተሳታፊዎችን ለመለየት ልዩ የመለያ ቁጥር ስለምንጠቀም ስሞትን መንገር አስፈሊጊ አይደለም። በዚህ ጥናት መሳተፋችሁ ርህራሄን ያማከለ የጤና ባለሙያዎች ህክምና ኣሰጣጥ እንቅስቃሴ ላይ ከፍተኛ አስተዋፅኦ ኣለዉ፡፡ በዚህ ጥናት ላይ በመሳተፍዎ ምንም አይነት ጥቅምም ሆነ ክፍያ አይኖርም፡፡ ነገር ግን በእያንዳንዱ ጥያቄ ላይ የእርስዎ ታማኝነት እና ትክክለኛ መልስ የጥናቱን አላማ ከግብ ለማድረስ ከፍተኛ ሚና ይኖረዋል፡፡. ለዚህ መጠይቅ ከ 10 እሰከ 20 ደቂቃዎች ወስዳችሁ በመመለሳቹ ለትብብራችሁ በጣም እናመሰግናለን፡፡ ከጥያቄዎቹ ወስጥ ግልፅ ያለሆነ ካለ በማንኛውም ሰዓት መጠየቅ ትችላላችሁ፡፡

ለበለጠ መረጃ ወይም ማብራርያ ከፈለጉ የዚህ ጥናት ባለቤት በሚከተለው አድራሻ ማግኘት ይችላሉ፡፡

ስም፡መርከብ ዘርኣይ

ስ.ቁጥር፡+251976036236

ኢሜይል፡ merkebzeray@gmail.com

ለመቀጠል ፈቃደኛ ነዎት?

አዎ አይደለሁም

አዎ ካሉ በማመስገን መቀጠል

ካልተስማሙ በማመስገን ወደ ሌላ ተሳተፊ ይቀጥሉ

2: የስምምነት መግለጫ ቅፅ

ከላይ የተጠቀሰው መግለጫ ስለተረዳሁ ለመሰተፍ ፈቃደኛ ነኝ

ያረጋገጠው::

የተሳታፊ ፊርማ ____________________________ቀን______________________

የመረጃ ሰብሳቢ ሰም______________________________ፊርማ_________________

የቃለ-መጠይቁ መረጃ ቁጥር_________________

የጀመረበት ሰዓት____________ ያለቀበት ሰዓት___________

**አዲስ አባባ ዩኒቨርሲቲ**

**ጤና ሳይንስ ኮሌጅ**

**የማህበረ-ሰብ ጤና ትምህርት ቤት**

**የሆስፒታሉ ስም_____________________________________**

**ክፍል 1: ማህበራዊ እና የስነ- ህዝብ መረጃ**

**መመሪያ፡እባክዎን ቀጥሎ የቀረቡትን ጥያቄዎችና የተሰጡትን አማራጮች ድምፅዎን ከፍ አድርገው በማንበብ የተሳታፊዎቹን ትክክለኛ ምላሽ ያክብቡት።**

| ተ.ቁ. | ጥያቄ | የመልሶች ምድብ | ይለፉት |
| --- | --- | --- | --- |
| 101 | ፆታ | 1. ወንድ 2. ሴት |  |
| 102 | ዕድሜዎት ስንት ነው(በሙሉዓመት)? | _____________________ |  |
| 103 | የመጡበት ክልል | 1. ትግራይ 2. እፋር 3. ኣማራ 4. ኦሮሞ 5. ሶማሊ 6. ቤንሻንጉል ጉሙዝ 7. ደቡብ 8. ጋምቤላ 9. ሀረር 10. ኣዲስ ኣበባ 11. ድሬዳዋ |  |
| 104 | መኖርያዎ | 1. ከተማ 2. ገጠር |  |
| 105 | ብሄር |  |  |
| 106 | ሃይማኖትዎ ምንድነው? | 1. ኦርቶዶክስ 2. እስልምና 3. ፕሮቴስታንት 4. ካቶሊክ 5. ሌላካለ(ይገለጽ) ___ |  |
| 107 | የጋብቻ ሁኔታ? | 1. ያላገባች 2. ያገባች 3. የተፋታች 4. ተለያይታ የምትኖር 5. የትዳር አጋር የሞተባት |  |
| 108 | የትምህርት ደረጃዎን ይግለፁ? | 1. ማንበብ እና መፃፍ ኣልችልም 2. ማንበብ እና መፃፍ እችላለዉ 3. ከ 1-6 ያጠናቀቀ/ች 4. ከ 7-12 ያጠናቀቀ/ች 5. ዲፕሎማ እና ከዛ በላይ |  |

**ክፍል2: የታኪሚዎቹ የህክምና ሁኔታ**

**መመሪያ፡ በዚህ ክፍል የቀረቡት ጥያቄዎች የዚህ ጥናት ተሳታፊ ህሙማን ስለህመማቸው፣ ሆስፒታል ስለመግባታቸው ፣ እና ስለተደጋጋሚ የሆስፒታል ጉብኝታቸው ዝርዝር መረጃ በመሰብሰብ ላይ ያተኩራል። ይህንን ጥያቄ ይዝለሉት በሚለው ላይ ትልቅ ትኩረት በማድረግ አባክዎ የዚህን ጥናት ተሳታፊዎች ቀጥሎ የቀረቡትን ጥያቄዎች ይጠይቋቸው።**

| ተ.ቁ. | ጥያቄ | የመልሶች ምድብ | ይለፉት |
| --- | --- | --- | --- |
| 201 | ታካሚዉ የተገኘበት ቦታ | 1. ተኝቶ ታካሚ 2. ተመላላሽ | መልሱ 2 ከሆነ ወደ ጥያቄ ቁጥር 206 ያምሩ። |
| 202 | የታካሚው ህመም ሙሉ መጠርያ |  | እባክዎትን ከካርድ ይመለከቱ። |
| 203 | የታካሚው የህክምና ዙር |  | እባክዎትን ከካርድ ይመለከቱ። |
| 204 | ለምን ያህል ጊዜ ሆስፒታል ገብተው ታክመዋል? (በቀናትይገለፅ) | __________________ |  |
| 205 | አሁን ሆስፒታል ገብተው ህክምና የጀመሩበትን ጨምሮ ከዚህ በፊት ለምን ያህል ጊዜ ሆስፒታል ገብተው ታክመዋል? | _________________ |  |
| 206 | ህክምና ከጀመሩበት ጊዜ ጀምሮ ( የዛሬውን ጨምሮ) ለምን ያህል ጊዜ ተመላልሰው ታክመዋል? | _____________________________ | አልጋ የያዘ ታካሚ ከሆነ ወደ ጥያቄ ቁጥር 301 ያምሩ። |
| 207 | ከዚህ በፊት በጥቁር አንበሳ ሆስፒታል ገብተው ታክመው ያውቃሉ? | 1. አዎ አውቃለሁ 2. ኣላውቅም | መልሱ “አላውቅም” ከሆነ ወደ ጥያቄ ቁጥር 301 ያምሩ። |
| 208 | ከዚህ በፊት ለመጨረሻ ሆስፒታል በገቡበት ጊዜ ለምን ያህል ቀናት በሆስፒታል ቆዩ? | __________________ |  |

**ክፍል ሶስት፡ ርህራሄ ተኮር የህክምና አገልግሎት ሰጪዎችን አስመልክቶ የታካሚዎች ግምገማ**

**መመሪያ፡ቀጥሎ ለቀረቡት ጥያቄዎች አስቀድሞ የቀረቡትን ዝርዝር መግለጫዎች ለተሳታፊዎች ካነበቡላቸው በህዋላ ዶክተሩ ወይም ሌላ የጤና ተንከባካቢው ያደረጉትን ርህራሄ ተኮር ህክምና ከአንድ ጀምሮ እስከ አስር ማርክ በመስጠት ይግለፁ።**

|  | የርህራሄ እንክብካቤ የሚያካትቱ ነገሮች | የምላሽ ደረጃ | | | | | | | | | | ይታለፍ |
| --- | --- | --- | --- | --- | --- | --- | --- | --- | --- | --- | --- | --- |
|  |  | 1 | 2 | 3 | 4 | 5 | 6 | 7 | 8 | 9 | 10 |  |
| ቀጥሎ በዶክተሮች፣ ነርሶች ና የጤና ተንከባካቢ ባለሙያዎች በህሙማን እና በቤተሰቦቻቸው መሃከል ሊኖር የሚገባውን ግንኙነት ለማሻሻል ስለሚደረገው የርህራሄ ተኮር ህክምና አቀራረብ እወስድዎታለሁ። ዋና አላማው ህሙማን (ታካሚዎች) ከዶክተሮች፣ ነርሶች ና ከጤና ተንከባካቢ ባለሙያዎች ሊያገኙት የሚገባውን ርህራሄ እንዲሁም መግባባት ለማሻሻል የታለመ ነው።  የሚያክምዎ/ የምታክምዎ ዶክተር ለእርስዎ ያላቸው ርህራሄ እና እንክብካቤ እጅግ አስደሳች ከሆነ አስር ማርክ ይሰጣሉ። ርህራሄው ና እንክብካቤው ዝቅ ያለ ከሆነ እንደ ደረጃው ከአስር ዝቅ ያለ ማርክ ይሰጣሉ። እንድ ማርክ ሰጡ ማለት ርህራሄ እና እንክብካቤ ሙሉ በሙሉ የጎደላቸው ናቸው ማለትዎ ነው። | | | | | | | | | | | | |
| 301 | ዶክተሩ፣ ነርሷ፣ የጤና ባለሙያው ለእርስዎም ፣ ለቤተሰቦችዎም፣ ለቅርብ አጋሮችዎም አክብሮት ይሰጣል /ትሰጣለች። |  |  |  |  |  |  |  |  |  |  |  |
| 302 | እርስዎ በደምብ ሊረዱት በሚችሉት መልኩ ሁኔታዎችን በማብራራት ይገልፅልዎታል /ትገልፅልዎታለች። |  |  |  |  |  |  |  |  |  |  |  |
| 303 | የምርመራ ውጤትዎን በተገቢው ሰአት ና ፍጥነት ይሰጥዎታል/ ትሰጥዎታለች። |  |  |  |  |  |  |  |  |  |  |  |
| 304 | ሃኪምዎ የሚመለከትዎት በሽተኛ አድርጎ ሳይሆን አክብሮት ሊሰጥዎት እንደሚገባሰው ነው። |  |  |  |  |  |  |  |  |  |  |  |
| 305 | እርስዎ የሚናገሩትን በአግባቡ ያዳምጣል / ታዳምጣለች። |  |  |  |  |  |  |  |  |  |  |  |
| 306 | ሃኪምዎ የህክምና ውሳኔ የሚሰጠው / የምትሰጥዎት እርስዎን ካማከረ / ካማከረች በህዋላ ነው። |  |  |  |  |  |  |  |  |  |  |  |
| 307 | በሃኪምዎ ላይ ሙሉ በሙሉ እምነት አለዎት። |  |  |  |  |  |  |  |  |  |  |  |
| 308 | ሃኪምዎ ህመምዎ በራስዎ ላይ፣ በቤተሰቦችዎ ና አጋሮችዎ ላይ ጫና እንደሚፈጥር በደምብ ይረዳል። |  |  |  |  |  |  |  |  |  |  |  |
| 309 | ሃኪምዎ ስጋትዎን፣ የውስጣዊ ስሜቶችዎን ና የሚያስጨንቅዎትን ነገሮች ሁሉ በነፃነት እንዲያካፍሉት ያደርጋል። |  |  |  |  |  |  |  |  |  |  |  |
| 310 | ሃኪሙ/ሃኪሟ እርስዎ ላሉበት ሁኔታ ተገቢ ትኩረት፣ እንክብካቤ ና ርህራሄ ይሰጣሉ። |  |  |  |  |  |  |  |  |  |  |  |
| 311 | ሀኪሙ/ ሃኪሟ ከእርስዎ ጋር በቂ ጊዜ ያሳልፋሉ። |  |  |  |  |  |  |  |  |  |  |  |
| 312 | ሃኪሙ/ ሃኪሟ ውስጣዊ ስሜትዎን ለመረዳት ይጥራሉ። |  |  |  |  |  |  |  |  |  |  |  |
